# Refractory Thyroid Eye Disease Unresponsive to Teprotumumab: A Case Report

**DOI:** 10.7759/cureus.48861

**Published:** 2023-11-15

**Authors:** Gurdeep Singh, Brittany Taylor, Samantha Michalek

**Affiliations:** 1 Endocrinology, Diabetes and Metabolism, Our Lady of Lourdes Memorial Hospital, Binghamton, USA; 2 Family Medicine, Our Lady of Lourdes Memorial Hospital, Binghamton, USA

**Keywords:** nonresponse, proptosis, euthyroid graves disease (egd), teprotumumab, thyroid eye disease (ted)

## Abstract

Thyroid eye disease (TED) is a complex autoimmune condition that can cause proptosis, ophthalmoplegia, diplopia, optic nerve compression, and vision loss. These clinical findings are caused by a complex pathological mechanism characterized by thyroid-stimulating hormone receptor autoantibodies activating thyroid-stimulating hormone receptors (TSH-Rs). Overexpressed insulin-like growth factor 1 (IGF-1) receptors found in orbital fibroblasts form complexes with these TSH-Rs, leading to the inflammation and expansion of these tissues. Teprotumumab, a human monoclonal antibody sold under the brand name Tepezza, is currently the only FDA-approved immunotherapy for the treatment of TED. Given as an intravenous infusion every three weeks, teprotumumab works by suppressing IGF-1 receptors, thereby interfering with TSH-R and IGF-1 complex-mediated actions in these fibroblasts. The efficacy of teprotumumab was established in randomized, placebo-controlled clinical trials, which demonstrated clinically meaningful improvements in proptosis, inflammation, and diplopia. While teprotumumab has been shown to be efficacious, our patient with TSHRAb-positive euthyroid thyroid-associated ophthalmopathy who presented with diplopia did not have any significant improvement following the standard treatment dose of eight infusions over a 24-week period. This case underscores not only barriers to treatment, such as the high cost of teprotumumab but also highlights the importance of identifying risks for nonresponse.

## Introduction

Thyroid eye disease (TED), also called Graves’ ophthalmopathy or Graves’ orbitopathy, is an autoimmune-mediated inflammatory disease most commonly associated with Graves’ disease. The prevalence of patients with Graves’ disease who will be affected by thyroid-associated ophthalmopathy is around 25-40% [1]. Perros et al. [2] reported an estimated prevalence of 10/10,000 among the general European population; however, the overall incidence of TED is not well defined. Although rare, euthyroid ophthalmopathy has a global prevalence of 7.9% [3]. Historically, euthyroid patients develop milder ophthalmic symptoms and have better responses to treatment. Although euthyroid Graves' disease (EGD) and Graves' disease share multiple similarities with regard to pathogenesis, they are different in that EGD is specific to an autoimmune response in the eye and the orbit, while in Graves' disease, there is also activation of receptors on the thyroid gland [4]. In general, TED is caused by a complex pathological mechanism characterized by thyroid-stimulating hormone receptor autoantibodies activating thyroid-stimulating hormone receptors (TSH-Rs). Overexpressed insulin-like growth factor 1 (IGF-1) receptors found in orbital fibroblasts form complexes with these TSH-Rs, leading to the inflammation and expansion of these tissues [5]. Those at greatest risk for developing TED include females and smokers [6], as well as individuals with a history of radioiodine therapy [7], high levels of TSH-R autoantibodies [8], and a high serum cholesterol level [9].

The clinical presentation of TED ranges from dryness to increased intraocular pressure and optic nerve compression, leading to loss of vision. Patients most frequently present complaining of excessive tearing, redness, photophobia, pain behind the eyes, and ocular discomfort. Ophthalmic manifestations include eyelid retraction, periorbital edema, erythema, chemosis, proptosis, and restrictive strabismus leading to diplopia [10-12]. The severity of symptom presentation is classified via four primary grading systems: the Clinical Activity Score (CAS), NO SPECS Classification system, European Group on Graves’ Orbitopathy (EUGOGO) scale, and VISA system with the latter being the two most currently used [13]. The EUGOGO scale categorizes TED severity as a mild, moderate-to-severe, or very serious disease. The mild disease classification describes cases with mild exophthalmos, mild eyelid retraction, and limited extraocular muscle (EOM) involvement, with treatment being relatively conservative. Moderate-to-severe TED cases present with restricted eye motility, significant exophthalmos, and a greater degree of inflammatory changes. Treatment at this stage generally requires escalation to biological infusion therapy and/or systemic steroids. The most severe EUGOGO scale categorization includes conditions such as compressive optic neuropathy and corneal ulceration, which would require surgical intervention [12]. The VISA classification system focuses on TED symptoms and physical exam findings by assessing four independently graded parameters: vision (1 point), inflammation (10 points), strabismus (6 points), and appearance (3 points). A graded score out of 20 is assigned based on the results. In this model, the inflammatory index component is primarily used to guide treatment. Patients with a score of less than 4 are treated conservatively, whereas a score greater than 5/10 may require a more aggressive treatment regimen, as this is suggestive of progressive inflammation [11].

Teprotumumab, a human monoclonal antibody sold under the brand name Tepezza, is currently the only FDA-approved immunotherapy for the treatment of TED. Current guidelines recommend the use of this biologic in patients with moderate-to-severe TED who present with significant proptosis and/or diplopia when there is no response to initial treatment with glucocorticoids [14]. Given as an IV infusion every three weeks, teprotumumab works by suppressing IGF-1 receptors, thereby interfering with TSH-R and IGF-1 complex-mediated actions in these fibroblasts [15]. The efficacy of teprotumumab was established in two randomized, placebo-controlled clinical trials, which both demonstrated a clinically meaningful improvement in proptosis, inflammation, and diplopia. Phase III study participants had a 78% overall response when treated with teprotumumab, as compared to only 7% in the placebo group [16], with comparable therapeutic effect in the prior phase II study [17]. Due to the rarity of EGD in the euthyroid, current literature studying the clinical responsiveness to teprotumumab is exclusively limited to populations with Graves’ disease. There is no evidence at this time to support alternative management for patients with euthyroid ophthalmopathy as compared to those with hyperthyroidism. Of note, however, Douglas et al. reported that both the experimental arm and control arm in this phase III multicenter study represented Graves’ disease patients with treatment-induced euthyroidism [16].

Here, we present a unique case of TED in which the patient had normal thyroid function, but significant Graves’ orbitopathy, which was refractory to teprotumumab.

## Case presentation

A 64-year-old Caucasian male with a history of depression, anxiety, and tobacco use consulted our endocrinology office for elevated PTH 217.3 pg/mL (normal range: 14.0-97.0 pg/mL) with hypercalcemia 11.1 mg/dL (normal range: 8.4-10.4) and was subsequently diagnosed with primary hyperparathyroidism. Three months later, the patient underwent right inferior parathyroidectomy and his PTH level normalized.

On initial presentation, the patient also reported a six-month history of gradually worsening double vision which improved with unilateral occlusion. Associated symptoms included bilateral eye pain, swelling, irritation, tearing, and mucopurulent discharge. His ocular examination was remarkable for esotropia in primary gaze and minimal exophthalmos with no resistance to retropulsion. His eyelid retraction was evaluated using the margin reflex distance, measuring about 5.5 to 6.0 mm in each eye. Ocular motility was notable for -2 limitation of both abduction and elevation bilaterally. Examination also revealed 1+ injection of the conjunctiva and edema of both the upper and lower eyelids. The pupillary reaction was normal in both eyes.

Initial endocrinology workup was performed according to relevant clinical guidelines (Table 1). Despite his physical presentation, serologic studies were not consistent with a diagnosis of Graves’ disease. The patient was ultimately diagnosed with TSHRAb-positive euthyroid thyroid-associated ophthalmopathy and referred to ophthalmology.

**Table 1:** Thyroid function test results at presentation.

	Lab Parameters	Reference Range
TSH	0.56	0.36-3.74 mIU/mL
Free T4	0.90	0.70-1.70 ng/dL
Total T3	0.84	0.58-1.59 ng/mL
TSHR-Ab	4.26	<1.75 IU/L

**Figure 1:**
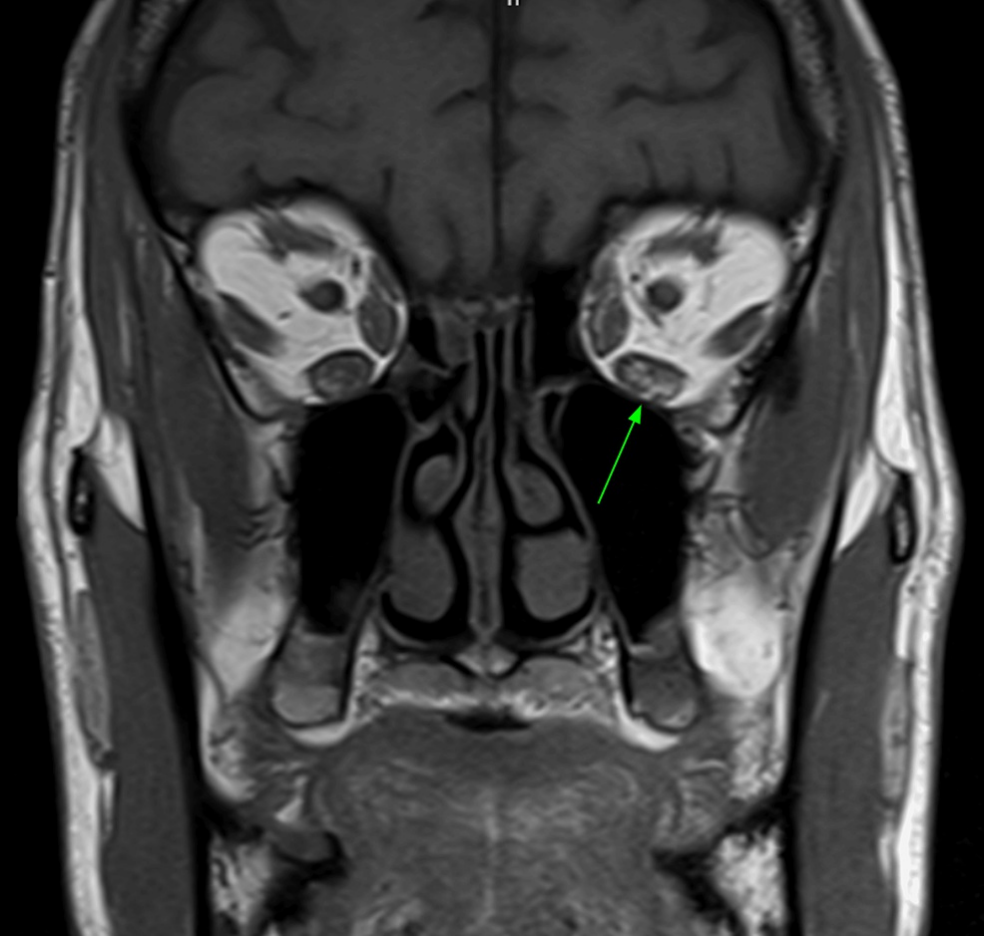
MRI, T1-weighted image, showing bilateral fatty infiltration of the inferior recti muscles, more prominent in the left eye (green arrow). Marginal intraocular muscle enlargement can also be seen in the bilateral inferior recti and lateral recti muscles.

The patient was initially treated by ophthalmology with a four-week course of once-daily 20 mg oral prednisone with no improvement in his clinical presentation. The low-dose steroid was discontinued after one month and he started receiving the standard eight-cycle treatment of teprotumumab infusions at three-week intervals. After completing one full round of treatment with teprotumumab, the patient showed minimal improvement in his condition. He noted some improvement in his eye pain, swelling, irritation, tearing, and discharge; however, the diplopia and esotropia remained unchanged. An MRI was obtained for the evaluation of persistent diplopia to rule out any alternative condition that may have been mimicking thyroid-associated orbitopathy. His imaging demonstrated the characteristic findings of extraocular muscle enlargement with the appearance of T1 hyperintense fatty infiltration, ultimately supporting a diagnosis of TED (Figure 1).

At present, the patient still suffers from constant debilitating diplopia. He continues to have a large angle esotropia with left hypotropia. Alignment has now been stable for nearly two years and the oculofacial plastic and reconstructive surgery specialist is recommending bilateral medial rectus and left inferior rectus recession surgery in an effort to improve his ocular deviation and diplopia. As an alternative, he was advised on the potential benefits of re-treatment with an additional round of teprotumumab. Ultimately, the patient felt that the cost of teprotumumab was disproportionately high when compared to its minimal success in the treatment of nonresponsive TED patients.

## Discussion

TED is a complex autoimmune condition that can cause proptosis, ophthalmoplegia, double vision, optic nerve compression, and vision loss [18]. TED typically has an acute inflammatory phase, known as the active phase, followed by a chronic fibrotic phase, or inactive phase [19]. The active phase is characterized by immune-mediated inflammation and expansion of orbital tissues, which are in turn responsible for the ocular manifestations of TED [20]. The inactive phase represents a stabilization of fibrotic changes, which grossly restricts movement of the eyes. At that point, rehabilitative surgery is generally considered to be the only viable therapy [19]. All other management strategies should be implemented during the active phase of the disease [21]. In this case, we report on the inadequate response to medical management of a euthyroid patient who presented with typical signs and symptoms of active phase TED.

The ability of corticosteroids to reduce active-phase inflammation has made this therapy a well-established and long-standing, first-line treatment in the management of moderate to severe TED. Furthermore, the literature suggests that IV corticosteroids are more effective and better tolerated than oral steroids [22]. Despite documented cases of euthyroid TED patients being successfully treated with IV corticosteroids [23], there is a possibility for symptom relapse. This was studied by Bartalena et al., who reported that after 24 weeks of treatment with IV steroids, 21% of patients experienced a relapse in symptoms [24].

Orbital decompression surgery for TED is typically reserved for cases of dysthyroid optic neuropathy (DON), where there is a significant threat of permanent vision loss. This is performed by the removal of either orbital fat or bone to enlarge the orbit and decompress the compromised structures. However, restorative surgical treatments to manage proptosis, restrictive strabismus, and eyelid retraction are also carried out in the moderate to severe inactive phase of TED [25]. In the present case, diplopia refractory to medical management led to a proposal from the ophthalmologist for surgical treatment, which the patient declined.

Additional possibilities for the management of TED include orbital radiotherapy and the antioxidant agent selenium, as well as biological agents such as rituximab, tocilizumab, and teprotumumab. Conservative management with once-daily supplemental selenium has been shown to slow disease progression in patients with mild TED [26]. Despite its questionable efficacy, radiotherapy continues to be utilized in the active phase of moderate to severe TED [14]. In one double-blind, randomized trial, Prummel et al. [27] saw an improvement in diplopia and extraocular motility for patients with mild ophthalmopathy after they received orbital irradiation. Conversely, a study performed by Gorman et al. [28] reported that orbital radiotherapy showed no significant therapeutic effect. The use of the anti-CD20 monoclonal antibody rituximab and the anti-IL-6 monoclonal antibody tocilizumab has also remained controversial. Although there is support for the clinical benefit of these biologic agents in moderate-to-severe TED [29-32], current literature is limited. Pérez-Moreiras et al. [33] presented convincing evidence to support the efficacy of intravenous tocilizumab in patients with TED refractory to intravenous steroids in one small, nonrandomized study (N=18). These results were later reproduced by the same group in a larger, randomized clinical trial (N=32) [34]; however, no large prospective study data has been published on the efficacy of tocilizumab, and it is not currently approved by the FDA for the treatment of TED.

Since its approval in January 2020 by the U.S. Food and Drug Administration (FDA), teprotumumab has remained the only biologic therapy designated for use in the treatment of TED [25] and is expected to be authorized in the future by the European Medicines Agency [14]. Teprotumumab is a monoclonal antibody antagonist to insulin-like growth factor-1 receptor (IGF-1R), which prevents stimulation of orbital fibroblast via interference with TSH-R and IGF-1 complex-mediated actions in these fibroblasts [35-37]. Although the mechanism is not completely understood, literature shows that teprotumumab works by downregulating the expression of IL-6 and IL-8, proinflammatory cytokines, which would normally be stimulated by TSH in thyroid-associated ophthalmopathy [15]. Phase II and phase III multicenter, double-masked, randomized, placebo-controlled clinical trials demonstrated that patients with active moderate-to-severe TED who were treated with teprotumumab had a marked improvement in symptoms. Both studies reported a reduction in proptosis of greater than 2 mm, and at least a 2-point reduction in their Clinical Activity Score (CAS), as compared to the placebo group. Of note, while patient populations in the RCTs were limited to active moderate-to-severe TED, the drug was approved by the FDA without specific reference to the disease stage [38]. Teprotumumab is given intravenously, with patients receiving eight weight-based infusions every three weeks. The first dose is 10 mg/kg, followed by 20 mg/kg doses for the remaining seven infusions [39]. Horizon Therapeutics, the biotechnology company responsible for marketing Tepezza, reported in 2020 that the cost of teprotumumab is approximately $14,900 per vial. Accounting for the weight-based dosing, the average patient weighing 70 kg would require 23 vials of the medication to complete a full course of treatment, equating to $342,700 for just one standard round of therapy [14].

In light of these prohibitive treatment costs, the ability of clinicians to predict who will be a nonresponder to teprotumumab is paramount. With limited therapeutic options and pivotal trial results strongly supporting the efficacy of teprotumumab compared to conventional approaches, guidelines for treatment with teprotumumab should remain relatively inclusive. Furthermore, in the more recent OPTIC-X study, an extension of the phase III trial, Douglas et al. [40] reported that 2/5 teprotumumab nonresponder patients from the initial study demonstrated subsequent improvement following re-treatment with eight infusions over 24 weeks.

## Conclusions

To conclude, clinicians should be aware of the possibility of refractory disease in TSHRAb-positive euthyroid thyroid-associated ophthalmopathy. Unfortunately, after receiving the standard treatment dose of eight teprotumumab infusions over a 24-week period, our patient showed only minimal symptomatic improvement. The literature discussing the factors that would cause teprotumumab to be ineffective is currently negligible. Future studies, ideally with a larger sample size, will be needed to identify patient populations in which a repeat treatment course of teprotumumab would be both cost-effective and clinically advantageous. While the exact cause of poor response to treatment is unknown at this time, this refractory case of TED suggests that further studies are warranted to help guide clinicians in choosing the management strategy, which will result in the best outcome for their patients.
